# Copy number variation of the SELENBP1 gene in schizophrenia

**DOI:** 10.1186/1744-9081-6-40

**Published:** 2010-07-08

**Authors:** Shirly Amar, Ofer Ovadia, Wolfgang Maier, Richard Ebstein, RH Belmaker, Dan Mishmar, Galila Agam

**Affiliations:** 1 Psychiatry Research Unit, Faculty of Health Sciences, Ben-Gurion University of the Negev, and Mental Health Center, Beersheva, Israel; 2 Department of Life Sciences and National Institute of Biotechnology (NIBN), Ben-Gurion University of the Negev, Beer Sheva, Israel; 3 Department of Psychiatry, University of Bonn, Bonn, Germany; 4 Department of Psychology, Hebrew University, Mt. Scopus and S. Herzog Memorial Hospital, Jerusalem, Israel

## Abstract

**Background:**

Schizophrenia is associated with rare copy-number (CN) mutations. Screening for such alleles genome-wide, though comprehensive, cannot study in-depth the causality of particular loci, therefore cannot provide the functional interpretation for the disease etiology. We hypothesized that CN mutations in the SELENBP1 locus could associate with the disorder and that these mutations could alter the gene product's activity in patients.

**Methods:**

We analyzed SELENBP1 CN variation (CNV) in blood DNA from 49 schizophrenia patients and 49 controls (cohort A). Since CN of genes may vary among tissues, we investigated SELENBP1 CN in age- sex- and postmortem interval-matched cerebellar DNA samples from 14 patients and 14 controls (cohort B). Since CNV may either be *de-novo *or inherited we analyzed CNV of the SELENBP1 locus in blood DNA from 26 trios of schizophrenia probands and their healthy parents (cohort C). SELENBP1 mRNA levels were measured by real-time PCR.

**Results:**

In cohort A reduced CN of the SELENBP1 locus was found in four patients but in none of the controls. In cohort B we found reduced CN of the SELENBP1 locus in two patients but in none of the controls. In cohort C three patients exhibited drastic CN reduction, not present in their parents, indicating *de-novo *mutation. A reduction in SELENBP1 mRNA levels in the postmortem cerebellar samples of schizophrenia patients was found.

**Conclusions:**

We report a focused study of CN mutations in the selenium binding-protein1 (SELENBP1) locus previously linked with schizophrenia. We provide evidence for recurrence of decreased CN of the SELENBP1 locus in three unrelated patients' cohorts but not in controls, raising the possibility of functional involvement of these mutations in the etiology of the disease.

## Background

Genomic deletion, duplication and insertion, designated copy number variation (CNV), are found in all human beings, could be either common, rare or de novo [1] and may influence gene expression and phenotypic variation [2-6]. CNV have been linked to the pathogenesis of both monogenic and complex disorders [7-13] including brain-related disorders such as schizophrenia [14-17]. Specifically, twenty five percent of subjects carrying a 3-Mb microdeletion in chromosome 22q11.2 exhibited psychiatric manifestations including autism, attention-deficit hyperactivity disorder (ADHD) and schizophrenia [18]. Recent whole genome CNV screens revealed significant association of rare CNVs in several loci with schizophrenia [16,19-21]. Confirmed *de novo *CNV mutations were significantly associated with schizophrenia and were about eight times more frequent in sporadic (but not in familial) cases of schizophrenia as compared to unaffected controls [21]. These findings suggest that CNVs in certain loci are more prevalent in schizophrenia patients as compared to controls.

Chromosome 1q21.3 harbors known CNVs in the general population [3,5,22,23]. Interestingly, the 1q21-22 region has been reported to be linked with schizophrenia with a likelihood of linkage (LOD) score of 6.5 (Brzustowicz and others, 2000). The gene encoding selenium binding protein (SELENBP)1 which has been mapped to this region has recently been reported to be associated with schizophrenia [24]. Moreover, SELENBP1 mRNA levels were up-regulated in postmortem brain and in blood cells from schizophrenia patients [25,26]. Indeed, selenium is implicated in neuroprotection [27-32] and selenium deficiency has been reported to be associated with increased schizophrenia rates [33]. We therefore hypothesized that CNV including the SELENBP1 locus is associated with schizophrenia. To this end we compared blood DNA from schizophrenia patients with healthy controls originating from the same ethnicity group. Since CNV varies among tissues [34,35] we then studied SELENBP1 CNV in postmortem cerebellum of schizophrenia patients and unrelated matched controls. Among other brain regions the cerebellum has also been implicated as involved in the pathophysiology of schizophrenia [36,37]. Since CNV may either be *de novo *or inherited [1] we further analyzed CNV of the SELENBP1 gene in trios of schizophrenia probands and their healthy parents in whole blood DNA. In order to find out whether CNV in the SELENBP1 locus associates with the previously reported up-regulation of brain SELENBP1 in schizophrenia, we measured SELENBP1 mRNA levels in the same postmortem cerebellar specimens studied for CNV.

## Materials and methods

### Patient cohorts

Discovery cohort: Blood DNA samples from 49 unrelated schizophrenia patients and 49 healthy controls originated from the Bedouins of the Negev population. The samples from the patient group and from the normal controls were collected for separate previously described studies after informed consent [38-40]. The original protocols were approved by the Helsinki committee (Beer-Sheva). Another cohort was age- sex- and postmortem interval-matched cerebellar samples from 14 schizophrenia patients and 14 matched normal controls were obtained from the Victoria Brain Tissue Repository at the Mental Health Research Institute of Victoria, Melbourne, Australia. Their detailed demographic data are summarized in Table 1. Permission to carry out studies was obtained from the Ethics Committee of the Victoria Institute of Forensic Medicine and the North Western Mental Health Program Behavioural and Psychiatric Research and Ethics Committee. An unrelated replication cohort of 78 blood DNA samples from 26 unrelated schizophrenia patients and their healthy parents originated from Israeli Arabs recruited for previously described studies after informed consent [41,42]. The original protocol was approved by the Helsinki committee (Beer-Sheva). All samples of the three cohorts became de-identified prior to our study.

**Table 1:** Postmortem brain cohort's demographic data

*Schizophrenia patients*							*Control subjects*				
Age	Sex	PMI	pH	DoI	CoD	Neuroleptics	Age	Sex	PMI	pH	CoD
38	m	36.00	6.07	11	Hanging	FD (25 mg, 3 weekly)	35	m	35.00	6.13	Coronary artery atheroma
35	m	47.00	6.27	17	Perforated gastric ulcer	FD (50 mg, 3 weekly)	38	m	44.00	6.19	Coronary artery atheroma
47	m	41.50	6.52	21	Multiple injuries	Chlorpromazine (400 mg daily),	48	m	24.00	6.37	Coronary artery atheroma
53	m	43.00	6.23	7	Aspiration/food	Haloperidol (150 mg, 2 weekly)	53	m	12.00	6.34	Pulmonary thromboembolism
						*Chlorpromazine (200 mg daily)					
						LAST RECORDED 14/6/88, 6 yrs prior to death					
69	m	44.50	6.38	47	Ischaemic heart disease	Trifluoperazine HCl (5 mg, daily)	62	m	66.00	6.50	Acute myocardial infarct
61	m	37.50	6.46	38	Ischaemic heart disease	*FD (62.5 mg, 2 weekly)	68	m	69.00	6.59	Coronary artery atheroma
						LAST RECORDED 22/1/92, 4 yrs prior to death					
65	F	50.00	6.35	18	Ruptured abdominal aneurysm	FD (25 mg, 2 weekly),	66	F	43.00	6.37	Acute myocardial infarct
						Haloperidol (5 mg, daily)					
57	m	24.00	6.06	28	Coronary artery atheroma	*FD (18.75 mg, 3 weekly),	57	m	27.00	6.43	Ischaemic heart disease
						LAST RECORDED 29/1/93, 3 yrs prior to death					
69	m	48.00	6.44	6	Carbon monoxide poisoning	*Haloperidol (7 mg, daily)	68	m	41.00	6.06	Aortic stenosis
						LAST RECORDED 26/11/91, 5 yrs prior to death					
23	m	78.00	6.19	5	Multiple injuries	*Haldol (150 mg, 4 weekly),	22	m	51.00	6.58	Exsanguination
						LAST ADMINISTERED 3 weeks prior to death					
26	m	52.00	6.39	2	Carbon monoxide poisoning	*Haldol (75 mg, 2 weekly),	25	m	35.00	6.15	Right ventricular hypertrophy
						LAST ADMINISTERED 2 days prior to death					
38	F	20.00	6.43	17	Burning	FD (31.25 mg, 2 weekly)	36	F	60.00	6.40	Dilated cardiomyopathy
41	m	35.00	6.64	15	Hanging	*FD (25 mg, ? weekly)					
						LAST RECORDED 15/11/88, 9 yrs prior to death					
47	F	50.00	6.31	20	Pneumonia	*Respiridone (12 mg, daily),	47	F	24.00	5.89	Pulmonary embolus
						LAST PRESCRIBED 6 months prior to death	32	F	56.00	6.16	Coronary artery atheroma

PMI = postmortem interval (hrs); DoI = duration of illness (yrs); CoD = cause of death;

### DNA purification

DNA for the dertermination of CNV was extracted from whole blood using standard techniques (Phenol-chloroform). DNA concentration and quality was quantified using a Nanodrop ND-1000 spectrophotometer (Nanodrop Technologies, Wilmington, DE). DNA concentration was brought to 50 ng/μl for the SELENBP1 amplification and to 5 ng/μl for the Factor 8 (F8, a gene locus lacking CNV) amplification.

### RNA purification and cDNA synthesis

For the determination of SELENBP1 mRNA levels RNA purification, determination of purity and concentration and cDNA synthesis were carried out as previously described [43].

### Measurement of DNA copy number by real time PCR

Quantitative PCR was performed using the F8 gene locus as a reference for normalization of the real time PCR experiments. This gene was chosen since it does not exhibit CNV [44]. F8 forward (CTACCATCCAGGCTGAGGTTTATG) and reverse (CACCAACAGCATGAAGACT GACA) primers were used as previously described [44]. A fragment of 254 bp of exon 12 of the SELENBP1 gene was amplified using the forward (CCACCAGGGAAGGCTCTGTGA) and reverse (GGGTGCCAAGAGAGAGCAGAA) primers designed using the program OLIGO and based on the published DNA sequence in Genbank (NM_003944).

Real-time quantitative PCR was performed using Rotor Gene 3000 (Corbett Research, Sydney, Australia). Reactions were carried out in a final volume of 25 μl consisting of either 50 ng/μl for the SELENBP1 amplification or 5 ng/μl for Factor 8 amplification, 250 nM forward and reverse primers each and 12.5 μl ABsolute QPCR SYBR Green Mix (ABgene, Surrey, UK) including dNTPs, Taq DNA polymerase and reaction buffer. Real-time PCR assays of SELENBP1 and F8 CNV included an initial step of 15 min at 95°C to activate Taq polymerase, followed by 40 cycles comprising denaturing at 95°C for 15 sec, annealing at 58.8°C for 20 seconds and extension at 72°C for 15 sec. The reaction was concluded with 10 minutes 72°C as a final extension step. The fluorescence of the accumulating product was acquired for each cycle after an additional 5 seconds melting step at 78°C. Fluorescence monitoring occurred at regular intervals during the annealing phase and continuously throughout the melting phase. The cycle at which fluorescence exceeded background, the corrected threshold cycle (*C*_t_), is negatively related to the initial copy number in a given DNA sample. In order to assess the relative copy numbers of SELENBP1 each of the runs included a calibration curve of both F8 and SELENBP1 using a DNA pool which included four samples having the highest Ct values. The F8 calibration curve was generated using DNA concentrations of 1 × 10^9^, 10^7^, 10^5^, 10^4 ^and 10^3 ^copies/μl. Quantification of each sample was obtained by interpolation of *C*_t _values from the generated standard curve using the Rotor-Gene software. Corrected Ct values were calculated as previously described [45] All assays included a no template negative control. All samples were run thrice. The presented results are the average of the three runs. Since F8 is located on chromosome X, *KC_ti _*values [45] of the women were corrected accordingly.

### Measurement of mRNA levels by RT-real time PCR

Quantitative RT-PCR was performed using the house-keeping gene β-actin as an internal standard. β-actin forward (TCCCTGGAGAAGAGCTACG) and reverse (GTAGTTTCGTGGAT GCCACA) primers and SELENBP1 forward (TCATCAGGGAAGGCTCTGTGA) and reverse (GGGTGCCAAGAGAGAGCAGAA) primers were designed using the computer program OLIGO based on the cDNA structure of the genes published in Genebank.

SELENBP1 and β-actin first strand cDNA synthesis was carried out in separate reactions. One μg RNA was reverse transcribed into cDNA with ReverseiT 1st Strand Synthesis Kit (ABgene, Surrey, UK) for 45 min at 42°C in a final 20 μl reaction volume containing 1 μg total cellular RNA. To obtain PCR results within the linear range of detection, the cDNA products were diluted 1:5 for both SELENBP1 and β-actin. Real-time quantitative PCR was performed using Rotor Gene 3000 (Corbett Research, Sydney, Australia). Reactions were carried out in a final volume of 25 μl consisting of 2 μl purified cDNA (SELENBP1 or β-actin), 250 nM each forward and reverse primers and 12.5 βl ABsolute QPCR SYBR Green Mix (ABgene, Surrey, UK) providing dNTPs, Taq DNA polymerase and reaction buffer. SELENBP1 and β-actin real-time PCR assays included an initial 15 min at 95°C step to activate Taq polymerase, followed by 40 cycles each comprising denaturation at 95°C for 15 sec, annealing at 58°C for 20 seconds, and extension at 72°C for 15 sec. The fluorescence of the accumulating product was acquired at each cycle after an additional 5 seconds melting step at 78°C. Each sample was run three times. Final quantification of the mRNA levels was carried out as previously described [46].

### Statistical analysis

In the CNV determination procedure normalized *C*_t _values were not normally distributed. We thus compared the distribution of SELENBP1 normalized *C*_t _values of schizophrenia patients in each of the three cohorts with their respective controls using Kolmogorov-Smirnov two-sample tests [47]. This nonparametric test is sensitive to differences in parameters such as location, dispersion and skewness between distributions [47]. Tests were performed using Systat 9.0 (Systat Software, Inc., CA, USA). As mentioned above, the third cohort was comprised of 26 triads of schizophrenia probands and their healthy parents. Each replicate of each family is designated "sample". A total of 78 samples (26 families × 3 replicates = 78 samples) were analysed using the program PRIMER v6 (PRIMER-E Ltd, Plymouth, UK). Each sample is represented as a point in a three dimentional space defined by the SELENBP1 normalized *C*_t _values of each of the three family members. We generated a dissimilarity matrix comprising of the Euclidean distances between all possible pairwise sample comparisons after applying a by total sample standardization [48]. Each of these dissimilarity coefficients represents the natural distance between two samples in the distribution of SELENBP1 normalized *C*_t _values among family members [48]. To explore the "natural groupings" of the 78 samples, we used a non-metric multi-dimensional scaling (MDS) [49]. Our aim was to represent the 78 samples as points in low-dimensional space (i.e., 2-d) such that the relative distances apart of all points are in the same rank order as the relative dissimilarities (or distances) of the samples as measured by the dissimilarly matrix described above. A two-dimensional space representation is considered excellent when the stress value is smaller than 0.05 [50]. To test for significant differences between families exhibiting *de novo *CNV of the SELENBP1 locus and those in which CNV was inherited we used an analysis of similarity test (ANOSIM) [48], analogous of the standard one-way analysis of variance (ANOVA), but which operates on the dissimilarity matrix described above.

Student's t-test was used to compare SELENBP1 mRNA levels (relative to β-actin) between patients and controls of the postmortem cerebellar cohort.

## Results

We assessed the variation in copy number of the SELENBP1 locus by real time PCR. Since there is no 'wild type' copy number of SELENBP1 to be used as a reference we normalized the Ct obtained from each sample by *C*_t _of Factor 8 of the coagulation system, a locus lacking CNV and hence expected to serve as a normalizing reference for SELENBP1 copy number. We performed 3 case-control comparisons of SELENBP1 CNV pattern. Cohort A: Blood DNA from 49 schizophrenia patients and 49 healthy controls from Israeli Bedouin origin. Since copy number of genes may vary among tissues, we further investigated (Cohort B) SELENBP1 copy number in age- sex- and postmortem interval-matched cerebellar DNA samples from 14 schizophrenia patients and 14 unrelated matched controls originating from the Australian population [51]. Since a CNV may either be *de novo *or be inherited we analyzed CNV of the SELENBP1 locus in blood DNA from 26 Israeli Arab trios of schizophrenia probands and their healthy parents (Cohort C) Schizophrenia-related differences in CNV of a specific locus have previously been reported either in a qualitative manner [52] or in a statistical supported approach [53]. Here we report our analysis in both ways.

**Figure 1:**
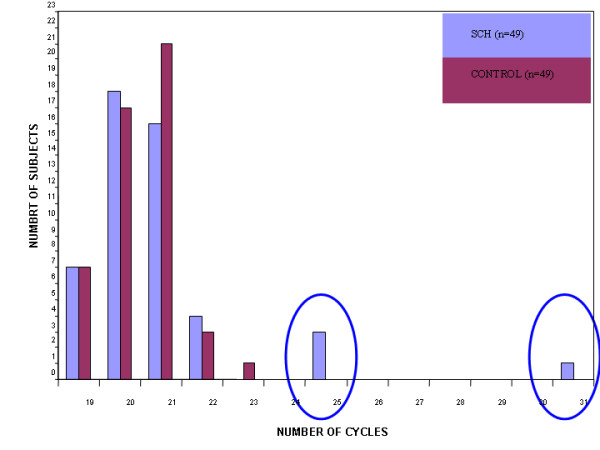
**A histogram of normalized cycle number required to reach a particular fluorescence threshold of the SELENBP1 gene in blood DNA of schizophrenia patients and healthy controls**. The distribution of the normalized *C*_t _values of SELENBP1 in the patient group is not significantly different from that of the control group (Kolmogorov-Smirnov two-sample test, p = 0.99). Circle denotes reduced copy number.

**Figure 2:**
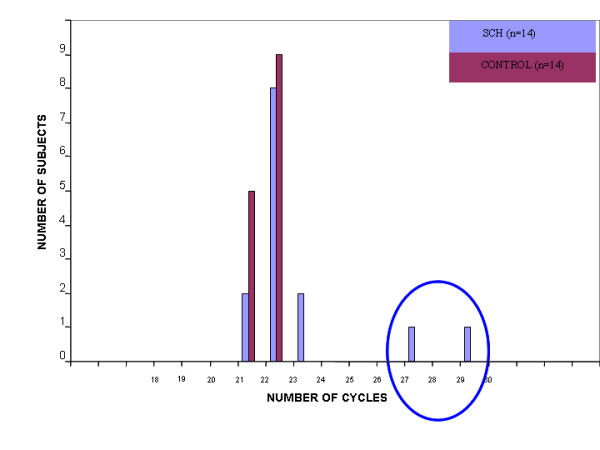
**A histogram of normalized cycle number required to reach a particular fluorescence threshold of the SELENBP1 gene in postmortem cerebellum DNA of schizophrenia patients**. The distribution of the normalized *C*_t _values of SELENBP1 in the patient group differed significantly from that of the control group (Kolmogorov-Smirnov two-sample test, p = 0.038). Circle denotes reduced copy number.

**Figure 3:**
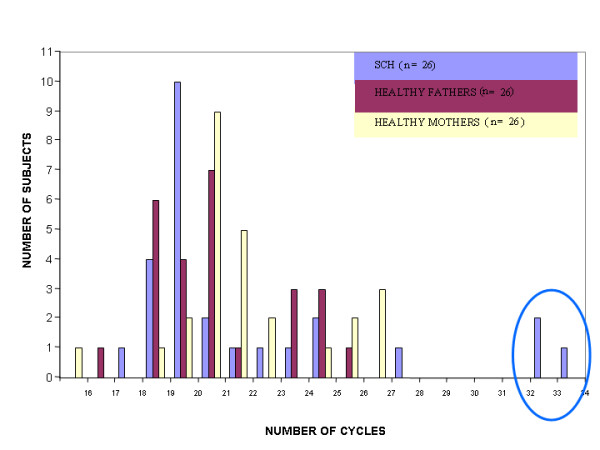
**A histogram of normalized cycle number required to reach a particular fluorescence threshold of the SELENBP1 gene in blood DNA of schizophrenia probands and their healthy partents**. The distribution of the normalized *C*_t _values of SELENBP1 in the patient group differed significantly from that of the mothers (Kolmogorov-Smirnov two-sample test, p = 0.012). Circle denotes reduced copy number.

**Figure 4:**
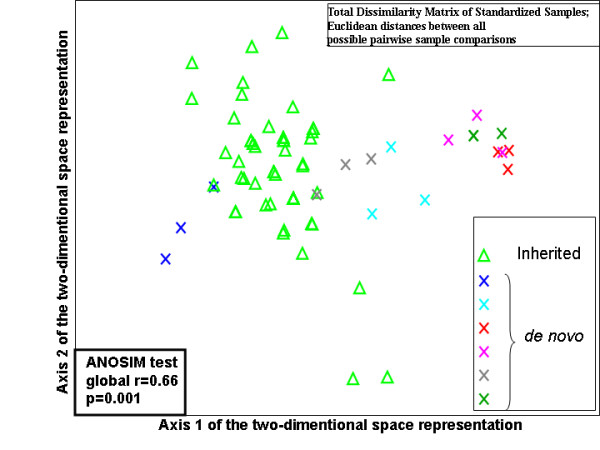
**Two-dimensional representation of non-metric multidimensional scaling (MDS) ordination of the triads cohort**. The cohort consisted of 78 samples (26 trios of schizophrenia probands and their health parents X 3 replicates = 78 samples). The analysis is based on the dissimilarity matrix generated by calculating the Euclidian distance between each of the possible pairwise sample comparisons. Stress value: 0.01; A two-dimensional space representation is considered excellent when the stress value is smaller than 0.05 [50]. ANOSIM test, Global r = 0.658, p = 0.001.

The distribution of the SELENBP1 normalized *C*_t _values characterizing the schizophrenia patients was right skewed in all three cohorts, indicating that the number of cycles required to reach the same threshold level was higher (lower copy number) among some of the schizophrenia patients in each of these three unrelated sample sets. In cohort A one schizophrenia patient exhibited drastic reduction and three patients had moderately reduced gene copy number of the SELENBP1 locus which were not found in any of the control subjects (Figure 1). This qualitative phenomenon did not reflect an overall significant difference in the distribution of normalized *C*_t _values of the SELENBP1 locus between the schizophrenia patients and the healthy controls. In the postmortem brain specimens (cohort B) a markedly reduced copy number of SELENBP1 in two schizophrenia specimens was observed (Figure 2). The distribution of the normalized *C*_t _values of SELENBP1 differed significantly between the patient and control groups (Kolmogorov-Smirnov two-sample test, p = 0.038). To discern between inherited and *de novo *CNV we analyzed the triads cohort (C) (Figure 3). The distribution of the normalized *C*_t _values of SELENBP1 in the patient group differed significantly from that characterizing the mothers (Kolmogorov-Smirnov two sample test, p = 0.012), but not the fathers (Kolmogorov-Smirnov two-sample test, p = 0.87). Three patients showed a drastically reduced copy number of SELENBP1, a phenomenon not observed in any of the healthy parents. Using an arbitrary difference of at least three normalized *C*_t _values between the patient and both of his/her parents as *de novo *CNV, overall, six families of the 26 exhibited *de novo *CNV of the SELENBP1 locus. These six families significantly differed from the other families (ANOSIM test, Global r = 0.658, p = 0.001) (Figure 4). Three of these families were those in whom the probands had the drastically reduced copy number of the SELENBP1 locus. Taken together, rare reduction in CNV of the SELENBP1 locus was found in patients but not in controls of the three studied cohorts.

SELENBP1 mRNA levels in the postmortem cerebellar specimens studied for CNV were about 16% significantly reduced between patients and controls [controls: 0.170 + 0.02 Arbitrary Units (AU); patients 0.143 + 0.02 AU; Student's t-test, n = 14 in each diagnostic group, t = -2.18, p = 0.037] with no correlation between SELENBP1 Ct (reflecting copy number) and mRNA levels in the patient group (r = -0.02).

## Discussion

Recent evidence supported the involvement of CNV in the etiology of complex disorders, in general, and in schizophrenia, in particular [10,16,19-21,53-55]. However, most of these reports deal with whole genome screens for CNV [16,19-21,54,55] and only few concentrate on specific loci [10,53]. The genome-wide assessment has the obvious advantage of providing concomitant evidence on multiple chromosomal loci. However, the results of the genome-wide studies [16,19-21] suggested that the concept of rare variants with high penetrance rather than the "common disease-common variant" hypothesis is more plausible to the etiology of schizophrenia [56]. Whereas association of common alleles could be identified by genome wide approaches, rare alleles are identified by methods which are not high throughput (such as re-sequencing of genes). When focusing on a certain candidate locus in a hypothesis-driven approach it is easier to identify rare alleles and the results obtained do not need statistical correction for multiple testing, possibly avoiding type-II error [56].

Here we present three replicating experiments demonstrating notable reduction in copy number of the SELENBP1 locus in nine of the patients of the three cohorts (n = 89) but not in the healthy controls (n = 115) in line with previous observations of a comparable extent of CNVs in this disorder [16,19]. Hence, rare reduction in SELENBP1 CNVs associates with schizophrenia providing proof of concept for our working hypothesis.

Most of the probands in our triads cohort exhibited CNV patterns similar to their healthy parents, implying inherited CNV. The latter is in line with other studies suggesting that most of the CNVs are stable [1,57]. However, six out of the 26 families exhibited *de novo *CNV of the SELENBP1 locus, in three of whom the probands had drastically reduced copy number. Patients with *de-novo *CNV differed significantly from the mothers but not from the fathers. It is noteworthy that new mutations are more common in sporadic cases of disease than in cases where patients have family history of the disease [58]. Since the reduced copy number was totally attributed to *de novo *mutations in the triads it is tempting to speculate that the same phenomenon underlies the reduced copy number of the SELENBP1 locus among the patients in the two other sample sets. This assumption is supported by Xu et al [21] who showed that confirmed *de novo *CNV mutations are significantly associated with schizophrenia.

CNV of a gene is among possible mechanisms for a difference in its function [1,59,60]. The right-skewed distribution reflecting reduced copy number of the SELENBP1 locus in the postmortem cohort correlated with 16% reduction of SELENBP1 mRNA levels of this cohort. This result is in contrast with the previously reported elevated mRNA levels of this gene in postmortem frontal cortex of schizophrenia patients. The discrepancy may reflect differences in CNVs among tissues [34,35] as well as documented tissue-specificity of SELENBP1 mRNA expression [61-63]. Although SELENBP1 mRNA levels of the two patients with the drastically reduced copy number of the gene's locus were below the mean of the patients' group, no linear positive correlation between the copy number and mRNA levels was obtained. It is possible that other modes of association between SELENBP1 CNV and its expression exist (e.g. a U-shaped relationship) since balanced CNVs with no functional effect in a carrier might create genomic instability only in future generations [64].

## Conclusions and limitations

We show that the SELENBP1 locus which is present in variable copy number in the general human population exhibits reduced copy number in schizophrenia patients from three different cohorts. Our cohorts are of a small size but the replication in three different cohorts of two different tissues compensate for this limitation. Although no correlation was found between the copy number change and the expression level of SELENBP1 it is important to note that only part of SELENBP1 exon 12 was studied and therefore the borders of the SLENEBP1-associated CNV are yet to be determined. Since this CNV could encompass a much larger genomic region it is possible that other factors would be affected by the copy number reduction. The reduced copy number in only 10% of the patients implies that other factors are associated with the disease in most schizophrenia cases. The general phenomenon of chromosomal instability in brain disorders [16,21,65-67], especially deletions in schizophrenia [10,19,20], raises the possibility that genome integrity tends to be less protected in this disorder. If this is true multiple regions could be prone to copy number change in schizophrenia, which is in line with the multifactorial nature of the disease. Indeed, elevated induction of common fragile sites, which are hot spots for chromosomal changes [68,69] have been reported in schizophrenia patients [70,71]. Taken together, our study underlines replicated association of reduced copy number of a given locus with schizophrenia. Further studies towards identifying candidate factors in this locus to contribute to the schizophrenia phenotype are warranted.

## Competing interests

The authors declare that they have no competing interests.

## Authors' contributions

SA designed and carried out the experiments and prepared the first draft of the manuscript as part of her PhD thesis. OO instructed SA in the statistical analysis. WM, RE and RHB collected the cohorts of the blood samples sponsored by their grant from the Deutsche Forschungsgemeinschaft. RHB was also involved in the initiative to study CNV in schizophrenia. DM and GA instructed SA during her PhD studies. GA and SA selected SELENBP1 as the target gene for the study. All authors read and approved the final manuscript.
